# Genetic Variation for Seed Metabolite Levels in *Brachypodium distachyon*

**DOI:** 10.3390/ijms20092348

**Published:** 2019-05-11

**Authors:** Yoshihiko Onda, Komaki Inoue, Yuji Sawada, Minami Shimizu, Kotaro Takahagi, Yukiko Uehara-Yamaguchi, Masami Y. Hirai, David F. Garvin, Keiichi Mochida

**Affiliations:** 1 Bioproductivity Informatics Research Team, RIKEN Center for Sustainable Resource Science, 1-7-22 Suehiro-cho, Tsurumi-ku, Yokohama, Kanagawa 230-0045, Japan; cprall427@gmail.com (Y.O.); komaki.inoue@riken.jp (K.I.); minami.shimizu@riken.jp (M.S.); kotaro.takahagi@riken.jp (K.T.); yukiko.uehara@riken.jp (Y.U.-Y.); 2 Kihara Institute for Biological Research, Yokohama City University, 641-12 Maioka-cho, Totsuka-ku, Yokohama, Kanagawa 244-0813, Japan; 3 Metabolic Systems Research Team, RIKEN Center for Sustainable Resource Science, 1-7-22 Suehiro-cho, Tsurumi-ku, Yokohama, Kanagawa 230-0045, Japan; yuji.sawada@riken.jp (Y.S.); masami.hirai@riken.jp (M.Y.H.); 4 Graduate School of Nanobioscience, Yokohama City University, 1-7-29 Suehiro-cho, Tsurumi-ku, Yokohama, Kanagawa 230-0045, Japan; 5 Plant Science Research Unit, United States Department of Agriculture, Agricultural Research Service, 1991 Upper Buford Circle, St. Paul, MN 55108, USA; david.garvin@ars.usda.gov; 6 Institute of Plant Science and Resource, Okayama University, 2-20-1 Chuo, Kurashiki, Okayama 710-0046, Japan; 7 Microalgae Production Control Technology Laboratory, RIKEN Baton Zone Program, RIKEN Cluster for Science, Technology and Innovation Hub, 1-7-22 Suehiro-cho, Tsurumi-ku, Yokohama, Kanagawa 230-0045, Japan

**Keywords:** *Brachypodium distachyon*, metabolome, QTL analysis, recombinant inbred line, vitamin B6, chrysanthemin

## Abstract

Metabolite composition and concentrations in seed grains are important traits of cereals. To identify the variation in the seed metabolotypes of a model grass, namely *Brachypodium distachyon*, we applied a widely targeted metabolome analysis to forty inbred lines of *B. distachyon* and examined the accumulation patterns of 183 compounds in the seeds. By comparing the metabolotypes with the population structure of these lines, we found signature metabolites that represent different accumulation patterns for each of the three *B. distachyon* subpopulations. Moreover, we found that thirty-seven metabolites exhibited significant differences in their accumulation between the lines Bd21 and Bd3-1. Using a recombinant inbred line (RIL) population from a cross between Bd3-1 and Bd21, we identified the quantitative trait loci (QTLs) linked with this variation in the accumulation of thirteen metabolites. Our metabolite QTL analysis illustrated that different genetic factors may presumably regulate the accumulation of 4-pyridoxate and pyridoxamine in vitamin B6 metabolism. Moreover, we found two QTLs on chromosomes 1 and 4 that affect the accumulation of an anthocyanin, chrysanthemin. These QTLs genetically interacted to regulate the accumulation of this compound. This study demonstrates the potential for metabolite QTL mapping in *B. distachyon* and provides new insights into the genetic dissection of metabolomic traits in temperate grasses.

## 1. Introduction

Cereal grains provide important nutrients in the human diet [1,2]. Therefore, grain quality is an agronomically important trait influenced by genetic factors, which are related to the metabolism of various phytochemicals in seeds [3]. Due to the growing awareness of the requirements for healthy foods for humans and livestock, the discovery of genes and alleles associated with metabolic phenotypes in cereal seeds has become a focus of breeding in order to generate more nutrient-rich cereals [4,5].

Metabolite quantitative trait locus (mQTL) analysis and metabolite-based genome-wide association studies (mGWAS) have enabled us to identify relationships between genetic and metabolic variation [6,7] and have led to advances in the discovery of genes involved in metabolic pathways and their regulatory networks [8,9,10]. In plants, these genetic approaches combined with high-throughput metabolomics have allowed researchers to discover genes associated with an abundance of metabolites in model plants, such as *Arabidopsis thaliana*, and in various crop species (such as *Beta vulgaris*, *Brassica napus*, *Brassica oleracea*, *Capsicum annuum*, *Cucumis melo* or *yza sativa*, *Prunus persica*, *Solanum lycopersicum*, *Solanum tuberosum*, *Triticum aestivum* and *Zea mays*) [7,11]. For example, in Arabidopsis, mQTL analysis [12] and mGWAS [13] identified several genes encoding enzymes that have roles in specific metabolic pathways. Similarly, in crop species, mQTL analysis [14] and mGWAS [15] have led to the identification of functional relationships between genes and metabolite accumulation. Thus, genetic approaches coupled with metabolomics-based phenotyping enables us to identify genetic factors involved in metabolic pathways and metabolite phenotypes. The model grass *Brachypodium distachyon* is a good model system for studying biological phenomena specific to temperate cereals as well as high-carbohydrate crops in the grass family due to its small size, short life cycle, self-fertility and fully sequenced diploid genome [16,17,18]. *B. distachyon* is phylogenetically close to crops within the tribe Triticeae, including wheat, which is one of the most important dietary sources of carbohydrates for humans [19]. Genetic tools, such as the TILLING platform [20,21], T-DNA insertion mutants [22,23] and a diverse collection of inbred lines [24,25], are available for *B. distachyon*. The *B. distachyon* lines exhibit wide phenotypic variation for many traits, including flowering time [26,27,28], root system architecture [29], resistance to barley stripe mosaic virus [30] and *Rhizoctonia solani* [31], water-use efficiency [32], freezing tolerance [33] and drought tolerance [34,35,36]. More recently, pan-genome sequencing highlighted the population structure and genomic variation across a diverse panel of lines [37]. We previously demonstrated metabolome-wide differences in the seeds and early vegetative stages between inbred lines of *B. distachyon* [38]. However, to our knowledge, this variation in metabolite abundance has not been exploited to identify the genetic factors associated with metabolite abundance in temperate grasses.

In this study, we performed a preliminary mQTL analysis on the accumulation of seed metabolites in *B. distachyon*. Using a widely targeted metabolome analysis of forty *B. distachyon* lines, we attempted to identify signature metabolites that displayed distinctive accumulation patterns across *B. distachyon* subpopulations. We also identified metabolites that differentially accumulated in seeds of the lines Bd21 and Bd3-1 before identifying QTLs linked with the variation in accumulated levels of 13 metabolites between these two lines. Our mQTL analysis further illustrated genetic factors that may regulate differences in the accumulation of 4-pyridoxate and pyridoxamine in the vitamin B6 metabolism pathway as well as the accumulation of chrysanthemin (Cyanidin 3-glucoside) in *B. distachyon* seeds. These findings demonstrate the feasibility of metabolome QTL mapping in *B. distachyon* and provide new insights into the genetic regulation of metabolite traits in temperate grasses.

## 2. Results

### 2.1. Genetic and Metabolic Variation among Forty B. distachyon Inbred Lines

**Figure 1:**
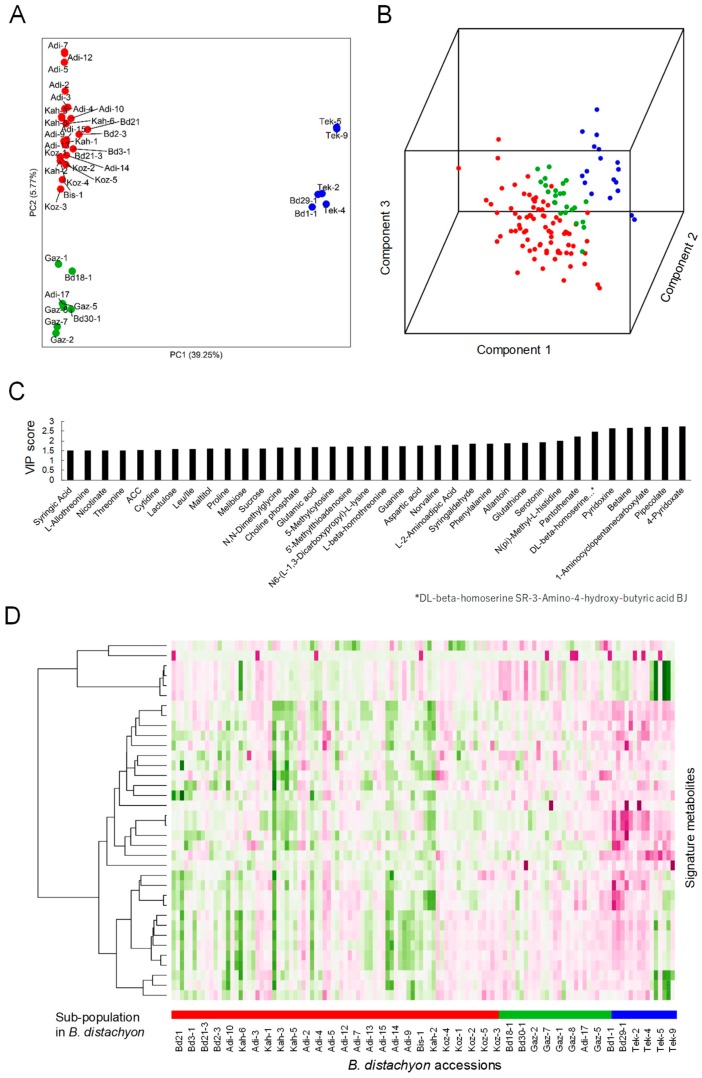
Genotype and metabolotype variation across forty *B. distachyon* inbred lines. (**A**) PCA plot of the forty natural *B. distachyon* lines based on the biallelic SNP dataset generated using publicly available GBS and whole-genome sequencing data. (**B**) PLS-DA plot of the forty *B. distachyon* lines based on accumulation profiles of the 183 metabolites in mature seeds. The colors for individuals are the same as in (**A**). (**C**) The signature metabolites based on the variable importance of projection (VIP) score (>1.5) of PLS-DA analysis. (**D**) Accumulation patterns of the signature metabolites across the forty *B. distachyon* lines. The heatmap represents hierarchically clustered accumulation patterns of the signature metabolites based on the normalized binary logarithm (log2) values of their quantitative data.

First, we identified 2564 biallelic SNPs across the forty lines based on their GBS and whole-genome sequencing datasets (Table S1) and verified their phylogenetic relationships. The principal component analysis (PCA) plot showed that they were classified into two major groups, one of which was further separated into two groups (Figure 1A). This was consistent with its estimated population structure based on a previous pan-genome analysis [37]. When we compared the accumulation patterns of 183 metabolites (Table S2) from these lines through PLS-DA analysis with the metabolotype datasets from our widely-targeted metabolome analysis, we found that the forty lines were again classified into two major groups, one of which could be further separated into two groups based on their metabolotypes. This suggests that the observed metabolotype variation reflected the population structure of *B. distachyon* (Figure 1B). To identify metabolites whose accumulation patterns were related to the population groups, we examined the variable importance of projection (VIP) scores based on the results of PLS-DA and identified thirty-six metabolites with VIP scores of >1.5 for at least one of the three components (Figure 1C). Specifically, 4-pyridoxate, DL-pipecolinic acid, 1-amino-1-cyclopentanecarboxylic acid, betaine and pyridoxine showed VIP scores of >2.5 for component 1, suggesting clear differences in accumulation patterns for these metabolites in relation to the metabolotypes associated with the stratified population groups of *B. distachyon*. 

### 2.2. Metabolotype Differences Between Bd21 and Bd3-1

**Figure 2:**
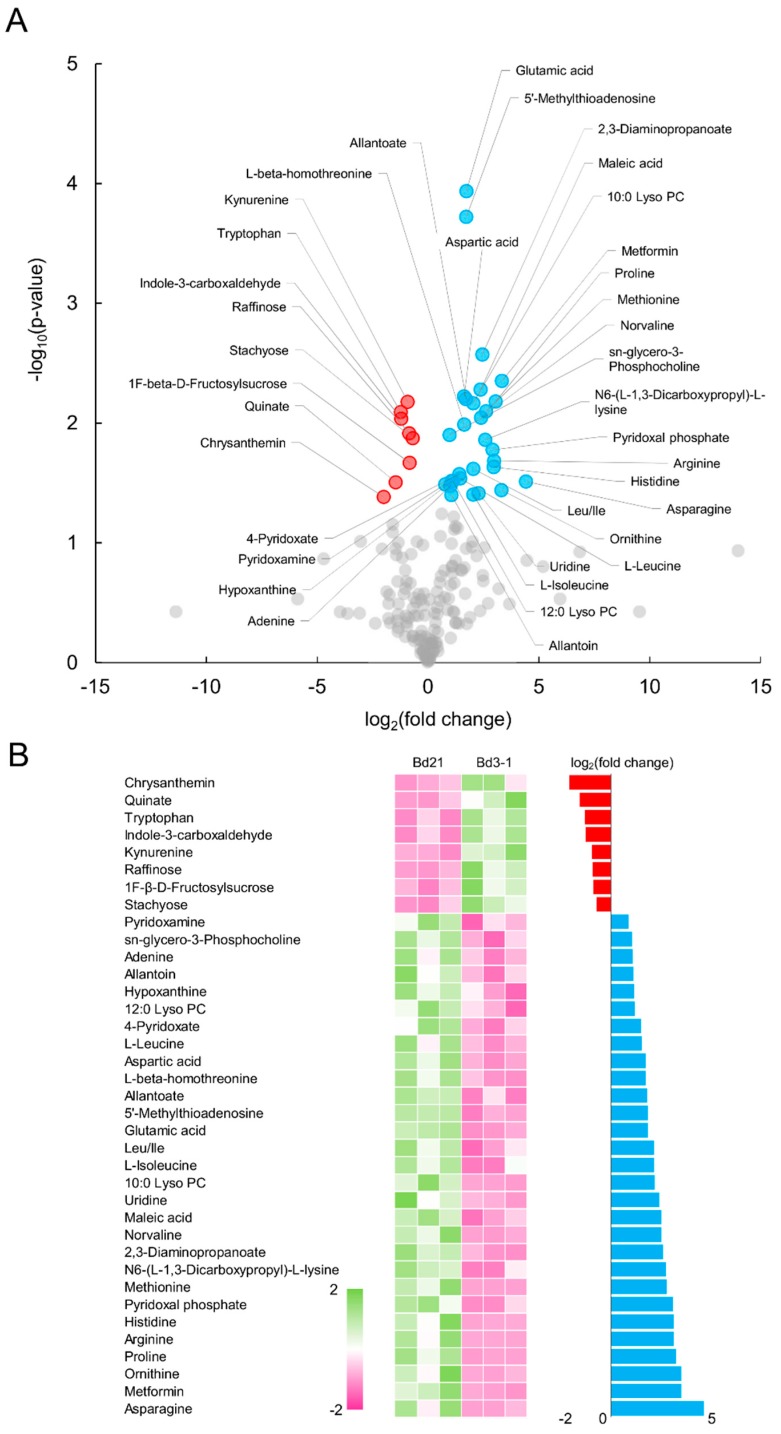
Differentially accumulating metabolites in Bd21 and Bd3-1 seeds. (**A**) Volcano plot representing metabolites in seeds between Bd21 and Bd3-1 (*p* < 0.05). (**B**) Accumulation patterns of thirty-seven differentially accumulating metabolites in Bd21 and Bd3-1 seeds as well as their fold change.

To identify genetic factors related to the differences in metabolite accumulation that were observed between the inbred lines from the same subpopulation and to link metabolic variation to genetic variation, we identified seed metabolites that differentially accumulated in Bd21 and Bd3-1, for which a recombinant inbred line (RIL) population exists [30]. We quantified 183 metabolites in mature Bd21 and Bd3-1 seeds and found that thirty-seven of these metabolites differentially accumulated (*p* < 0.05) between the two lines (Figure 2A). A total of twenty-nine and eight of those metabolites were more abundant in Bd21 and Bd3-1, respectively (Figure 2B, Table S3). We found differential accumulation patterns in a variety of amino acids between the two strains, which may have resulted from differences in the metabolic pathways related to amino acid biosynthesis. Moreover, we found differentially accumulating metabolites between the two lines that may be co-regulated through related metabolic pathways. Specifically, we found that Bd3-1 abundantly accumulated both tryptophan and kynurenine, an intermediate metabolite in the kynurenine pathway that is metabolized from tryptophan, which suggests variation in the metabolic regulatory networks of Bd21 and Bd3-1. In contrast, Bd21 abundantly co-accumulated pyridoxamine, 4-pyridoxate and pyridoxal phosphate, which are namely a compound of vitamin B6, a catabolite of vitamin B6 and the active form of vitamin B6, respectively. This suggests a difference in vitamin B6 metabolism between the two strains. These differentially accumulated metabolites represent distinct metabolic phenotypes between the closely related lines within a subpopulation of *B. distachyon*.

### 2.3. QTL Mapping of Metabolite Accumulation Using RILs Derived from a Cross Between Bd21 and Bd3-1

**Figure 3:**
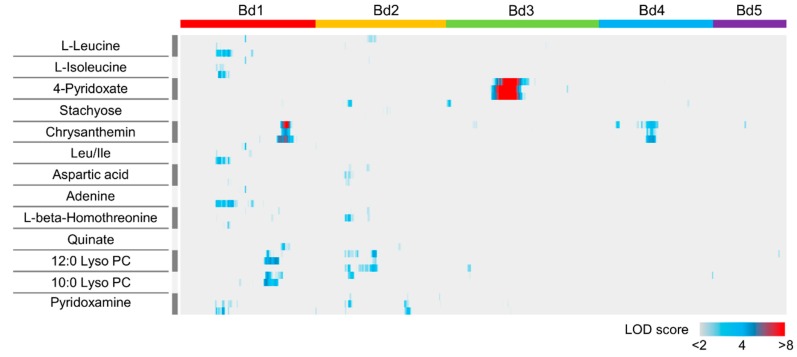
Results of mQTL analysis using an RIL population from the cross Bd3-1 × Bd21. Heat map representing the distribution of LOD scores for 17 metabolites across five chromosomes (Bd1, Bd2, Bd3, Bd4 and Bd5) in *B. distachyon*. The heat map for each metabolite is created using three replicates.

To identify genetic loci related to the metabolic phenotypes of the thirty-seven differentially accumulating metabolites in Bd21 and Bd3-1, we performed QTL mapping for each metabolite using an F_6_-derived RIL population derived by crossing those lines. After this, we reconstructed a linkage map based on 551 SNP markers distributed across the five *B. distachyon* chromosomes and previously used to genotype each RIL [30]. We found QTLs linked to thirteen metabolites that showed logarithm of the odds (LOD) scores higher than the threshold of 5% significance level between the two lines (Figure 3). Using our replicated metabolome measurements in the RIL population, we identified QTLs for the accumulation of chrysanthemin, catabolic products of vitamin B6 and the flavonoid anthocyanin, respectively (Figure 3). QTL analysis for the other differentially accumulating metabolites in the parental lines did not show well-defined or significant LOD peaks, suggesting that the underlying QTLs may have been composed of many genes with limited individual effects and/or that they may have strong interactions with environmental factors during seed maturation.

### 2.4. QTLs Related to Vitamin B6 Metabolism in B. distachyon

**Figure 4:**
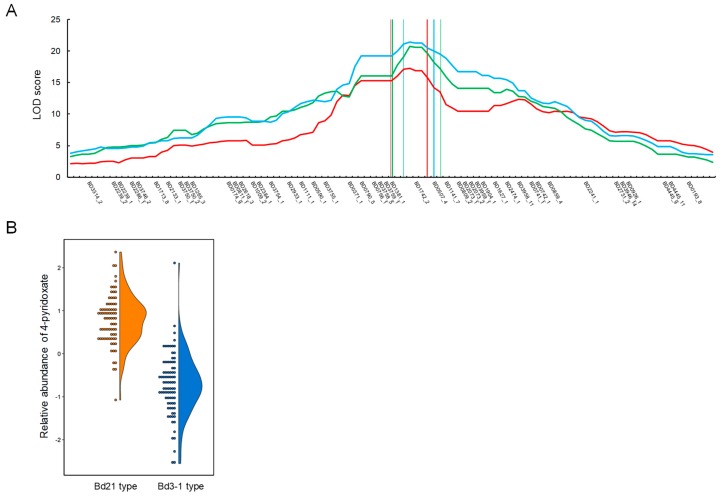
Genetic mapping of QTLs for 4-pyridoxate accumulation in the Bd3-1 × Bd21 RILs. (**A**) A QTL likelihood (LOD) map of the genomic region that covers the QTL region of interest, with a LOD significance threshold of *p* < 0.05. The colored LOD curves correspond to three replicates of metabolome measurements. (**B**) Average relative abundance of 4-pyridoxate in seeds for the genotypes nearest to the closest marker, BD1742_2, in the RILs.

From our metabolome phenotyping of the RIL population, we identified QTLs for the accumulation of metabolites in the vitamin B6 metabolism pathway that affect 4-pyridoxate and pyridoxamine on chromosomes 3 and 1, respectively (supported by 1000 permutation tests; α = 0.05) (Figure 3 and Table 1). We identified a single major QTL for the accumulation of 4-pyridoxate on chromosome 3 in the *B. distachyon* genome (Figure 4A). This was located near the SNP marker BD1742_2, for which the LOD score and percentage of variance explained by the QTL were large (LOD = 17.3–21.4 and 38.4–45.2%, respectively). These statistics, the clear co-segregation of the genotype from the closest marker and the accumulation of 4-pyridoxate across the RIL population (Figure 4B) suggest that the identified QTL was a major genetic factor regulating the differential accumulation of 4-pyridoxate in Bd21 and Bd3-1 seeds. We explored annotated genes in the chromosomal region flanked by the SNP markers BD1381_1 and BD0507_4, in which 284 genes were annotated in the Bd21 genome (Table S4). Additionally, we identified a QTL for the accumulation of pyridoxine on chromosome 1 with a threshold of α = 0.05 in 1,000 permutations. We found that the QTL was located near the SNP marker BD1723_1, for which the LOD score and percentage of variance explained by the QTL were LOD = 4.7 and 12.3%, respectively, suggesting a weak effect on the accumulation of pyridoxine. The results of interval mapping showed multiple LOD peaks on chromosome 1, 2 and 3, most of which were below the threshold (Figure 3). This suggests that the accumulation of pyridoxine may be regulated by multiple genes with weak effects. Using our metabolome analysis of Bd21 and Bd3-1, we found differences in the accumulation of pyridoxamine and 4-pyridoxate between the two lines (Figure 2) and our mQTL analysis illustrated that different genetic factors may regulate the accumulation level of these two compounds in vitamin B6 metabolism in *B. distachyon* seeds.

### 2.5. Interaction of QTLs for Accumulation of Chrysanthemin in B. distachyon

**Figure 5:**
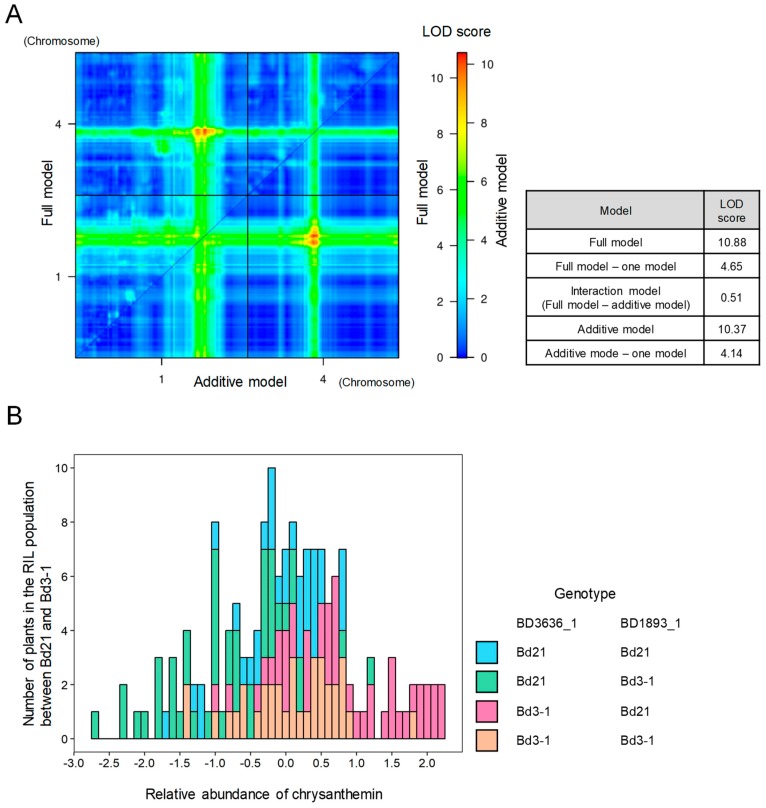
Interaction of QTLs in the accumulation of chrysanthemin in the Bd3-1 × Bd21 RILs. (**A**) LOD scores corresponding to additive or full (additive and interaction) models for each combination of chromosomes 1 and 4 QTLs for the accumulation of chrysanthemin. The upper left and lower right triangles represent the results of the full and additive models, respectively. The color scale indicates separate LOD score scales for the full and additive models. LOD scores computed using five models are represented on the two-dimensional QTL scan. (**B**) Combined effects of the two QTLs on the accumulation of chrysanthemin in the RILs. The histogram represents the phenotypic distribution of chrysanthemin accumulation in the RILs for the four genotype combinations corresponding to the two SNP markers Bd3534_1 and BD1893_1, located on chromosomes 1 and 4, with the maximum LOD score.

Using our metabolome QTL analysis, we found two QTLs for the accumulation of chrysanthemin on chromosomes 1 and 4 in the *B. distachyon* genome. We found that the QTL on chromosome 4 was located near the SNP markers BD3636_1 and/or BD4076_1, with LOD scores and percentage of variance explained by the QTL of LOD being 4.9–8.3 and 13.0–20.9%, respectively. The other QTL, positioned on chromosome 4, was located near BD1893_1 or BD3589_1, with LOD scores and percentage of variance explained by the QTL of LOD being 4.0–4.9 and 10.7%–12.9%, respectively (Table 2). By estimating the additive effects of these QTLs, we found that the QTLs on chromosomes 1 and 4 showed negative and positive effects on this metabolotype, respectively, and originated from Bd3-1 and Bd21, respectively. We explored annotated genes in the chromosomal regions of these QTLs flanked by the SNP markers BD0454_1–BD1404_2 and BD0833_2–BD4222_1 and found that 107 and 1120 genes are annotated in the Bd21 genome, respectively (Table S5). To assess the interactions between these two QTLs, we performed two-way interval mapping and calculated the likelihood under an additive QTL model and a full (additive + interaction) QTL model. We found a slight difference between the LOD scores of these two models (full model: 10.88 and additive model: 10.37) (Figure 5A), suggesting that they displayed additive effects rather than epistatic interactions for the accumulation of chrysanthemin in *B. distachyon* seeds. We also examined the distribution of allelic combinations for the closest SNP markers linked to the QTLs (BD3636_1 and BD1893_1) and the accumulation of chrysanthemin in the RIL population. We confirmed that the lines with allelic combinations of BD3636_1_Bd3-1_–BD1893_1_Bd21_ and BD3636_1_Bd21_–BD1893_1_Bd3-1_ showed more and less accumulation of chrysanthemin compared to other lines, respectively, further confirming the additive effect of the QTLs (Figure 5B). Thus, these findings represent a partial genetic dissection of chrysanthemin accumulation in *B. distachyon* seeds though mQTL mapping.

## 3. Discussion

After comparing the genetic population structure and metabolotypes of the forty *B. distachyon* inbred lines, our results suggested that seed metabolotypes may be stratified according to their subpopulations. Our multivariate analysis based on the metabolome dataset identified a list of signature metabolites whose accumulation patterns appear to represent specific characteristics in each of the *B. distachyon* subpopulations. International efforts focusing on genetic resource development for *Brachypodium* research [24,25] have contributed to an increase in the number of unique genotypes submitted to databases in various institutions. Due to its small genome size, small physical size and short life cycle, the large-scale exploration of genetic associations with phenotypic differences in *B. distachyon* will enable us to comprehensively identify genetic and environmental factors related to a variety of traits in the life cycles of temperate grass species. In addition to the pan-genome analysis in *Brachypodium distachyon* [37], the results from population-wide genotyping and population structure analyses will be a valuable resource for gene discovery through GWAS as it will describe the phenotypic differences found within a subpopulation [39]. Moreover, because the population structure is one of the major factors of genomic inflation in GWAS [40], QTL analysis with RIL populations will provide a useful complemental approach, especially for genetic analysis of phenotypic variations correlated with the population stratification. Therefore, the RIL populations across the subpopulations, such as Bd3-1/Bd1-1 [36] and Bd21/Bd1-1 [41], will provide us with opportunities to identify genetic factors associated with wide ranges of phenotypic variations. In addition to the recent advances in high-throughput genotyping [42] and plant phenotyping [43], widely targeted metabolomics can provide a useful analytical framework for chemical-based phenotyping to identify genes associated with metabolic phenotypes [6,7] in addition to providing metabolic insights into phenotypic plasticity and local adaptations mediated by chemical diversity in *B. distachyon* [24,25]. Moreover, such chemical diversity may be useful as an integrator to predict external phenotypes from genotypes [44].

Using a RIL population generated from a cross between Bd21 and Bd3-1, we performed an mQTL analysis that aimed to identify genetic factors related to the accumulation of several metabolites in *B. distachyon* seeds. Several previous studies have attempted mQTL identification in various plant species, which have found QTLs in 84 out of 181 metabolites (46.4%) in *A. thaliana* shoots [45], 58 out of 311 metabolites (18.6%) in *A. thaliana* seeds [46] and 453 out of 759 metabolites (59.7%) in rice grains [47]. In comparison to previous reports on mQTL identification, we identified a low number of QTLs in this present study (13 out of 37 metabolites that differentially accumulated between the parental strains; 35.1%). This may have been due to the small variation in the numbers of differentially accumulated metabolites found between the two strains as well as their genetic relatedness since both lines were collected in Iraq [48]. In this study, using our analysis of QTLs related to the accumulation of vitamin B6-related metabolites and the anthocyanin chrysanthemin, we demonstrated the effectiveness of this approach for exploring the genetic basis of the *B. distachyon* metabolome through our genetic dissection of metabolomics quantitative traits. Viewing the list of genes annotated in the QTL region of the accumulation of vitamin B6-related metabolites (Table S4), we found that the gene *Bradi3g23340* encodes a putative pyridoxamine 5′-phosphate oxidase (PPOX), which plays a role in vitamin B6 metabolism. Regarding the QTL region of the anthocyanin chrysanthemin, the list still contains many genes annotated on the QTL intervals (Table S5). Because further investigation is needed to determine the causal gene of the QTL, various genome resources developed in Brachypodium [20,23] will facilitate the process of genetically narrowing down the candidate genes and functional analysis through a detailed gene expression study and metabolome profiling during seed development and maturation process. Vitamins and anthocyanins are important nutritionally for both plants and humans [49,50,51,52]. Because vitamin B6 biofortification in staple crops has been shown to be a promising strategy for reducing the risk of vitamin B6 deficiency in human populations [53], understanding the genetic and chemical basis of vitamin B6 metabolism and phenotypic variation for its accumulation in cereal grains may improve our ability to successfully breed enhanced vitamin B6 levels. Other desired metabolites may be enhanced through similar strategies. Anthocyanins have also been a breeding target in staple crops, such as wheat, because of their benefits for human health [54]. Therefore, the genetic basis for the accumulation of these useful metabolites from *B. distachyon* will provide clues for the breeding of cool season crops, such as wheat, barley, rye and oat, in order to improve their nutritional content through comparative analysis between *B. distachyon* and these Triticeae crops.

## 4. Materials and Methods

### 4.1. Plant Materials

We used mature seeds from forty inbred lines of *B. distachyon* [24,25,55], whose genome-wide sequence datasets are available (whole genome sequencing datasets [37,56] or genotyping by sequencing datasets (Table S1). We used a recombinant inbred line (RIL) population derived from a cross between Bd3-1 (female) and Bd21 (male) [30] for our widely targeted metabolome analyses. We harvested mature seeds from these *B. distachyon* lines, which were grown under the conditions described previously [38].

### 4.2. Metabolome Analysis

We obtained quantitative data for metabolite accumulation using an ultra-performance liquid chromatography tandem/triple quadrupole mass spectrometer (Waters, Tokyo, Japan) as described previously [57]. For our metabolome analysis, we measured three independent samples from bulked seeds of the inbred lines and the RIL population. We replaced all missing values from the MS data area in the raw MS data with a value of 1 and divided the data area for each metabolite by the area of the internal standard and log 2-transformed for downstream analysis. We performed a partial least squares regression discriminant analysis (PLS-DA) of the processed MS data and calculated the variable importance in projection (VIP) score for each metabolite using the MetaboAnalyst 4.0 web interface with its auto-normalization (https://www.metaboanalyst.ca/, accessed on 27 Sep. 2018) [58].

### 4.3. Genetic Map Construction and QTL Analysis

We used the previously characterized SNPs [30] for genetic map construction in the Bd3-1 x Bd21 RIL population. A total of 551 SNP markers previously used to genotype the population were used to reconstruct a genetic map using the “plotMap” function after the conversion of the data type to RIL selfing using the “convert2riself” function and imputation of the missing genotypes using the “fill.geno” function of the R/qtl package “qtl” (version 1.40.8) [59]. Interval mapping was conducted using the “scanone” function of the R/qtl package with default parameters after the calculation of QTL genotype probabilities at a 1-cM step size across the genome using the “calc.genoprob” function. We used 1000 permutations to determine the genome-wide logarithm of the odds (LOD) threshold at a 5% significance level.

### 4.4. Genome-Wide SNPs

To examine the relationship between metabolite accumulation and genetic variation in the *B. distachyon* RIL population, we retrieved whole-genome sequences and genotyping-by-sequencing (GBS) datasets of forty *B. distachyon* inbred lines from the DDBJ sequence read archive (DRA) (https://www.ddbj.nig.ac.jp/dra/index-e.html, accessed on 14th September, 2018) (Table S1) and analyzed genomic sequence variation among the lines. The raw reads were trimmed using Trimmomatic (v0.36) [60] with the LEADING:20 TRAILING:20 MINLEN:50 commands. The trimmed reads were mapped to the *B. distachyon* Bd21 genome sequence retrieved from Phytozome (Bdistachyon_314_v3.0). Genome-wide SNPs were identified using the mpileup command of samtools [61].

### 4.5. Data Accessibility

Data on the metabolome analyses are available at Data Resources Of Plant Metabolomics (DROP Met), accessible via the Platform for RIKEN Metabolomics (PRIMe) website at http://prime.psc.riken.jp (accessed on 10 May 2019).

## Figures and Tables

**Table 1:** Summary of mQTLs related to vitamin B6 metabolism.

Metabolite	Replicate	Chromosome	QTLs (cM)	Maximum LOD	Percentage of Additive Effect (%)	Percentage of Variance Explained (%)	LOD Support Interval (1.5-LOD Dropping from the Maximum LOD)	Closest SNP Maker	Physical Location of Closest SNP Marker *
4-Pyridoxate	1	3	171.0	17.3	−62.8	38.4	168.3–173.0 cM (15.3–15.6)	BD1742_2	22,818,945
	2	3	171.0	20.7	−64.7	44.1	170.0–173.4 cM (19.1–18.2)		
	3	3	171.0	21.4	−67.2	45.2	168.3–174.0 cM (19.2–19.6)		
Pyridoxamine	3	1	108.4	4.7	−34.3	12.3	98.0–113.0 (2.8–2.7)	BD1723_1	30,380,964

* Physical location of SNP positions based on Cui et al. (2012) [30].

**Table 2:** Summary of mQTLs related to chrysanthemin accumulation.

Replicate	Chromosome	QTLs (cM)	Maximum LOD	Percentage of additive effect (%)	Percentage of Variance Explained (%)	LOD Support Interval (1.5-LOD Dropping from the Maximum LOD)	Closest SNP Maker	Physical Location of Closest SNP Marker *
1	1	262.0	8.3	45.6	20.9	258.0–268.0 (6.7–5.9)	BD3636_1	63,763,612
2	1	261.0	4.9	35.4	13.0	256.0–266.0 (3.4–3.1)	BD4076_1	63,379,714
3	1	261.0	6.2	39.5	16.1	242.0–265 (4.7–4.6)		
1	4	136.0	4.0	–33.3	10.7	122.0–146.0 (2.3–2.0)	BD1893_1	23,806,368
3	4	138.4	4.9	–35.7	12.9	127–141.7 (3.4–3.0)	BD3589_1	26,658,442

* Physical location of SNP positions based on Cui et al. (2012) [30].

## Supplementary Materials

Can be found at https://www.mdpi.com/1422-0067/20/9/2348/s1. Supplementary Table S1. Genome sequence datasets from the forty *B. distachyon* inbred lines analyzed in this study. Supplementary Table S2. Metabolome profiles of the 183 metabolites measured by widely-targeted metabolome analysis in seeds grains of the forty *B. distachyon* inbred lines. Supplementary Table S3. Metabolome profiles of the thirty-seven metabolites measured by widely-targeted metabolome analysis in seed grains of the *B. distachyon* Bd3-1 × Bd21 RILs. Supplementary Table S4. Genes annotated in the QTL region of 4-Pyridoxate content. Supplementary Table S5. Genes annotated in the QTL region of chrysanthemin content.

## References

[1] Bruinsma J. (2002). World Agriculture: Towards 2015/2030. Summary Report.

[2] McKevith B. (2004). Nutritional aspects of cereals. Nutr. Bulletin.

[3] Harrigan G.G., Martino-Catt S., Glenn K.C. (2007). Metabolomics, metabolic diversity and genetic variation in crops. Metabolomics.

[4] Bhullar N.K., Gruissem W. (2013). Nutritional enhancement of rice for human health: the contribution of biotechnology. Biotechnol. Adv..

[5] Yu S., Tian L. (2018). Breeding Major Cereal Grains through the Lens of Nutrition Sensitivity. Molecular. Plant.

[6] Luo J. (2015). Metabolite-based genome-wide association studies in plants. Curr. Opin. Plant. Biol..

[7] Hong J., Yang L., Zhang D., Shi J. (2016). Plant Metabolomics: An Indispensable System Biology Tool for Plant Science. Int. J. Mol. Sci..

[8] Keurentjes J.J.B., Fu J.Y., de Vos C.H.R., Lommen A., Hall R.D., Bino R.J., van der Plas L.H.W., Jansen R.C., Vreugdenhil D., Koornneef M. (2006). The genetics of plant metabolism. Nat. Genet..

[9] Saito K., Matsuda F. (2010). Metabolomics for functional genomics, systems biology and biotechnology. Annu. Rev. Plant. Biol..

[10] Davey J.W., Hohenlohe P.A., Etter P.D., Boone J.Q., Catchen J.M., Blaxter M.L. (2011). Genome-wide genetic marker discovery and genotyping using next-generation sequencing. Nat. Rev. Genet..

[11] Scossa F., Brotman Y., Lima F.D.E., Willmitzer L., Nikoloski Z., Tohge T., Fernie A.R. (2016). Genomics-based strategies for the use of natural variation in the improvement of crop metabolism. Plant Sci..

[12] Riewe D., Jeon H.J., Lisec J., Heuermann M.C., Schmeichel J., Seyfarth M., Meyer R.C., Willmitzer L., Altmann T. (2016). A naturally occurring promoter polymorphism of the Arabidopsis FUM2 gene causes expression variation and is associated with metabolic and growth traits. Plant J..

[13] Angelovici R., Lipka A.E., Deason N., Gonzalez-Jorge S., Lin H., Cepela J., Buell R., Gore M.A., Dellapenna D. (2013). Genome-wide analysis of branched-chain amino acid levels in Arabidopsis seeds. Plant Cell.

[14] Schauer N., Semel Y., Roessner U., Gur A., Balbo I., Carrari F., Pleban T., Perez-Melis A., Bruedigam C., Kopka J. (2006). Comprehensive metabolic profiling and phenotyping of interspecific introgression lines for tomato improvement. Nat. Biotechnol..

[15] Wen W.W., Li D., Li X., Gao Y.Q., Li W.Q., Li H.H., Liu J., Liu H.J., Chen W., Luo J. (2014). Metabolome-based genome-wide association study of maize kernel leads to novel biochemical insights. Nat. Commun..

[16] International Brachypodium I. (2010). Genome sequencing and analysis of the model grass Brachypodium distachyon. Nature.

[17] Bevan M.W., Garvin D.F., Vogel J.P. (2010). Brachypodium distachyon genomics for sustainable food and fuel production. Curr. Opin. Biotechnol..

[18] Girin T., David L.C., Chardin C., Sibout R., Krapp A., Ferrario-Mery S., Daniel-Vedele F. (2014). Brachypodium: a promising hub between model species and cereals. J. Exp. Bot..

[19] Shewry P.R., Hey S.J. (2015). The contribution of wheat to human diet and health. Food Energy Secur..

[20] Dalmais M., Antelme S., Ho-Yue-Kuang S., Wang Y., Darracq O., d’Yvoire M.B., Cezard L., Legee F., Blondet E., Oria N. (2013). A TILLING Platform for Functional Genomics in Brachypodium distachyon. PloS ONE.

[21] de Bang L., Torp A.M., Rasmussen S.K. (2018). TILLING in Brachypodium distachyon. Methods Mol. Biol..

[22] Thole V., Alves S.C., Worland B., Bevan M.W., Vain P. (2009). A protocol for efficiently retrieving and characterizing flanking sequence tags (FSTs) in Brachypodium distachyon T-DNA insertional mutants. Nat. Protoc..

[23] Hsia M.M., O’Malley R., Cartwright A., Nieu R., Gordon S.P., Kelly S., Williams T.G., Wood D.F., Zhao Y., Bragg J. (2017). Sequencing and functional validation of the JGI Brachypodium distachyon T-DNA collection. Plant J..

[24] Vogel J.P., Tuna M., Budak H., Huo N., Gu Y.Q., Steinwand M.A. (2009). Development of SSR markers and analysis of diversity in Turkish populations of Brachypodium distachyon. BMC Plant Biol..

[25] Tyler L., Fangel J.U., Fagerstrom A.D., Steinwand M.A., Raab T.K., Willats W.G., Vogel J.P. (2014). Selection and phenotypic characterization of a core collection of Brachypodium distachyon inbred lines. BMC Plant Biol..

[26] Schwartz C.J., Doyle M.R., Manzaneda A.J., Rey P.J., Mitchell-Olds T., Amasino R.M. (2010). Natural Variation of Flowering Time and Vernalization Responsiveness in Brachypodium distachyon. Bioenerg. Res..

[27] Ream T.S., Woods D.P., Schwartz C.J., Sanabria C.P., Mahoy J.A., Walters E.M., Kaeppler H.F., Amasino R.M. (2014). Interaction of Photoperiod and Vernalization Determines Flowering Time of Brachypodium distachyon. Plant Physiol..

[28] Bettgenhaeuser J., Corke F.M., Opanowicz M., Green P., Hernandez-Pinzon I., Doonan J.H., Moscou M.J. (2017). Natural Variation in Brachypodium Links Vernalization and Flowering Time Loci as Major Flowering Determinants. Plant Physiol..

[29] Pacheco-Villalobos D., Hardtke C.S. (2012). Natural genetic variation of root system architecture from Arabidopsis to Brachypodium: towards adaptive value. Philos. Trans. R. Soc. Lond. B Biol. Sci..

[30] Cui Y., Lee M.Y., Huo N., Bragg J., Yan L., Yuan C., Li C., Holditch S.J., Xie J., Luo M.C. (2012). Fine mapping of the Bsr1 barley stripe mosaic virus resistance gene in the model grass Brachypodium distachyon. PloS ONE.

[31] Kouzai Y., Kimura M., Watanabe M., Kusunoki K., Osaka D., Suzuki T., Matsui H., Yamamoto M., Ichinose Y., Toyoda K. (2018). Salicylic acid-dependent immunity contributes to resistance against Rhizoctonia solani, a necrotrophic fungal agent of sheath blight, in rice and Brachypodium distachyon. New Phytol..

[32] Des Marais D.L., Razzaque S., Hernandez K.M., Garvin D.F., Juenger T.E. (2016). Quantitative trait loci associated with natural diversity in water-use efficiency and response to soil drying in Brachypodium distachyon. Plant Sci..

[33] Colton-Gagnon K., Ali-Benali M.A., Mayer B.F., Dionne R., Bertrand A., Do Carmo S., Charron J.B. (2014). Comparative analysis of the cold acclimation and freezing tolerance capacities of seven diploid Brachypodium distachyon accessions. Ann. Bot..

[34] Luo N., Liu J., Yu X., Jiang Y. (2011). Natural variation of drought response in Brachypodium distachyon. Physiol. Plant.

[35] Fisher L.H., Han J., Corke F.M., Akinyemi A., Didion T., Nielsen K.K., Doonan J.H., Mur L.A., Bosch M. (2016). Linking Dynamic Phenotyping with Metabolite Analysis to Study Natural Variation in Drought Responses of Brachypodium distachyon. Front. Plant Sci..

[36] Jiang Y., Wang X., Yu X., Zhao X., Luo N., Pei Z., Liu H., Garvin D.F. (2017). Quantitative Trait Loci Associated with Drought Tolerance in Brachypodium distachyon. Front. Plant Sci..

[37] Gordon S.P., Contreras-Moreira B., Woods D.P., Des Marais D.L., Burgess D., Shu S., Stritt C., Roulin A.C., Schackwitz W., Tyler L. (2017). Extensive gene content variation in the Brachypodium distachyon pan-genome correlates with population structure. Nat. Commun..

[38] Onda Y., Hashimoto K., Yoshida T., Sakurai T., Sawada Y., Hirai M.Y., Toyooka K., Mochida K., Shinozaki K. (2015). Determination of growth stages and metabolic profiles in Brachypodium distachyon for comparison of developmental context with Triticeae crops. Proc. Biol. Sci..

[39] Wilson P.B., Streich J.C., Murray K.D., Eichten S.R., Cheng R., Aitken N.C., Spokas K., Warthmann N., Gordon S.P., Accession C. (2019). Global Diversity of the Brachypodium Species Complex as a Resource for Genome-Wide Association Studies Demonstrated for Agronomic Traits in Response to Climate. Genetics.

[40] Korte A., Farlow A. (2013). The advantages and limitations of trait analysis with GWAS: a review. Plant Methods.

[41] Woods D.P., Bednarek R., Bouche F., Gordon S.P., Vogel J.P., Garvin D.F., Amasino R.M. (2017). Genetic Architecture of Flowering-Time Variation in Brachypodium distachyon. Plant Physiol..

[42] Onda Y., Mochida K. (2016). Exploring Genetic Diversity in Plants Using High-Throughput Sequencing Techniques. Curr. Genom..

[43] Mochida K., Koda S., Inoue K., Hirayama T., Tanaka S., Nishii R., Melgani F. (2019). Computer vision-based phenotyping for improvement of plant productivity: a machine learning perspective. Gigascience.

[44] Handakumbura P.P., Stanfill B., Rivas-Ubach A., Fortin D., Vogel J.P., Jansson C. (2019). Metabotyping as a Stopover in Genome-to-Phenome Mapping. Sci. Rep..

[45] Lisec J., Meyer R.C., Steinfath M., Redestig H., Becher M., Witucka-Wall H., Fiehn O., Torjek O., Selbig J., Altmann T. (2008). Identification of metabolic and biomass QTL in Arabidopsis thaliana in a parallel analysis of RIL and IL populations. Plant J..

[46] Knoch D., Riewe D., Meyer R.C., Boudichevskaia A., Schmidt R., Altmann T. (2017). Genetic dissection of metabolite variation in Arabidopsis seeds: evidence for mQTL hotspots and a master regulatory locus of seed metabolism. J. Exp. Bot..

[47] Matsuda F., Okazaki Y., Oikawa A., Kusano M., Nakabayashi R., Kikuchi J., Yonemaru J.I., Ebana K., Yano M., Saito K. (2012). Dissection of genotype-phenotype associations in rice grains using metabolome quantitative trait loci analysis. Plant J..

[48] Garvin D.F., Gu Y.Q., Hasterok R., Hazen S.P., Jenkins G., Mockler T.C., Mur L.A.J., Vogel J.P. (2008). Development of genetic and genomic research resources for Brachypodium distachyon, a new model system for grass crop research. Crop Sci..

[49] Fitzpatrick T.B., Basset G.J., Borel P., Carrari F., DellaPenna D., Fraser P.D., Hellmann H., Osorio S., Rothan C., Valpuesta V. (2012). Vitamin deficiencies in humans: can plant science help?. Plant Cell.

[50] Mooney S., Hellmann H. (2010). Vitamin B6: Killing two birds with one stone?. Phytochemistry.

[51] Santos-Buelga C., Mateus N., De Freitas V. (2014). Anthocyanins. Plant pigments and beyond. J. Agric. Food Chem..

[52] Li D., Wang P., Luo Y., Zhao M., Chen F. (2017). Health benefits of anthocyanins and molecular mechanisms: Update from recent decade. Crit. Rev. Food Sci. Nutr..

[53] Fudge J., Mangel N., Gruissem W., Vanderschuren H., Fitzpatrick T.B. (2017). Rationalising vitamin B6 biofortification in crop plants. Curr. Opin. Biotechnol..

[54] Hong M.J., Kim D.Y., Nam B.M., Ahn J.W., Kwon S.J., Seo Y.W., Kim J.B. (2019). Characterization of novel mutants of hexaploid wheat (Triticum aestivum L.) with various depths of purple grain color and antioxidant capacity. J. Sci. Food Agric..

[55] Figueroa M., Alderman S., Garvin D.F., Pfender W.F. (2013). Infection of Brachypodium distachyon by formae speciales of Puccinia graminis: early infection events and host-pathogen incompatibility. Plos One.

[56] Onda Y., Takahagi K., Shimizu M., Inoue K., Mochida K. (2018). Multiplex PCR Targeted Amplicon Sequencing (MTA-Seq): Simple, Flexible and Versatile SNP Genotyping by Highly Multiplexed PCR Amplicon Sequencing. Front. Plant Sci..

[57] Sawada Y., Akiyama K., Sakata A., Kuwahara A., Otsuki H., Sakurai T., Saito K., Hirai M.Y. (2009). Widely targeted metabolomics based on large-scale MS/MS data for elucidating metabolite accumulation patterns in plants. Plant Cell Physiol..

[58] Chong J., Soufan O., Li C., Caraus I., Li S., Bourque G., Wishart D.S., Xia J. (2018). MetaboAnalyst 4.0: towards more transparent and integrative metabolomics analysis. Nucleic Acids Res..

[59] Broman K.W., Wu H., Sen S., Churchill G.A. (2003). R/qtl: QTL mapping in experimental crosses. Bioinformatics.

[60] Bolger A.M., Lohse M., Usadel B. (2014). Trimmomatic: a flexible trimmer for Illumina sequence data. Bioinformatics.

[61] Li H., Handsaker B., Wysoker A., Fennell T., Ruan J., Homer N., Marth G., Abecasis G., Durbin R., Genome Project Data Processing S. (2009). The Sequence Alignment/Map format and SAMtools. Bioinformatics.

